# Crisis in the gut: navigating gastrointestinal challenges in Gulf War Illness with bioengineering

**DOI:** 10.1186/s40779-024-00547-2

**Published:** 2024-07-08

**Authors:** Claudia A. Collier, Aelita Salikhova, Sufiyan Sabir, Steven Foncerrada, Shreya A. Raghavan

**Affiliations:** Department of Biomedical Engineering, Texas A&M University, College Station, TX 77843 USA

**Keywords:** Gulf War Illness, Bioengineering, Neuroimmune crosstalk, Gastrointestinal motility

## Abstract

Gulf War Illness (GWI) is characterized by a wide range of symptoms that manifests largely as gastrointestinal symptoms. Among these gastrointestinal symptoms, motility disorders are highly prevalent, presenting as chronic constipation, stomach pain, indigestion, diarrhea, and other conditions that severely impact the quality of life of GWI veterans. However, despite a high prevalence of gastrointestinal impairments among these veterans, most research attention has focused on neurological disturbances. This perspective provides a comprehensive overview of current in vivo research advancements elucidating the underlying mechanisms contributing to gastrointestinal disorders in GWI. Generally, these in vivo and in vitro models propose that neuroinflammation alters gut motility and drives the gastrointestinal symptoms reported in GWI. Additionally, this perspective highlights the potential and challenges of in vitro bioengineering models, which could be a crucial contributor to understanding and treating the pathology of gastrointestinal related-GWI.

## Background

From August 1990 to February 1991, the United States dispatched roughly 697,000 military personnel to the Persian Gulf for Operations Desert Shield and Desert Storm [1]. The war, despite its short duration of only 6 months, resulted in a lasting legacy of health issues for numerous soldiers who participated in the conflict. Approximately 25 – 35% of Gulf War combat veterans, in the months and years following the end of the war, began to exhibit a diverse array of symptoms of seemingly unknown origins [2–4]. This inexplicable disease was labeled broadly as Gulf War Illness (GWI) in reference to the shared experiences of Gulf War veterans afflicted by the disease. The increased complexity of GWI stems from various incursions into multiple body systems, including the central nervous system (CNS), the digestive, muscular, and respiratory systems, and largely the immune system, exacerbating physiological and psychological complications [4]. GWI is a multisymptomatic disorder encompassing fatigue, fibromyalgia, functional gastrointestinal disorders (FGIDs), neurological and psychological problems, sleep disturbances, and skin rashes [2, 5, 6]. Notably, FGIDs include symptoms of diarrhea, constipation, nausea, and irritable bowel disease (IBS) reported by GWI veterans [5, 7–9]. Neuroinflammation has been shown to be central to most of the reported symptoms [10–15] across multiple organ systems. Therefore, the gastrointestinal (GI) system and its connection to neuroinflammation via the enteric nervous system may play a pivotal role in GWI [15–17]. Gaining a comprehensive understanding of how neuroinflammation affects the GI tract and developing treatments that specifically target local enteric neuroinflammation could potentially lead to a significant breakthrough in effectively managing and treating GWI for our aging veterans.

GWI affects many biological systems of the body, but there is even greater complexity within the GI-related symptoms. The GI tract is comprised of multiple layers of control (including the central and peripheral nervous system, enteric nervous system, muscular system, and immune components) that work together to maintain physiological function. In GWI, GI dysfunction impacts these systems and leads to long-lasting effects on GI motility while driving enteric neuroinflammation [15, 16], even decades after the initial toxic exposure has been removed. Due to the intricate nature of both GI motility and GWI, there is currently no universally effective treatment available for all symptomatic manifestations. Treatments for general GI disorders encompass dietary and lifestyle modifications, probiotic supplementation, and fecal microbiota transplants. Despite these advancements, the exploration of treatment interventions specifically targeting gut health in veterans with GWI remains limited [18]. This review first explores potential GWI exposures, with an emphasis on prevalent GI symptoms in Gulf War veterans, and critically evaluates the effectiveness and limitations of existing therapeutic interventions aimed at alleviating specific GI symptoms. We provide a brief overview of the disparate systems within the GI tract that contribute to its essential function of GI motility, central to many symptomatic manifestations of GI dysfunction in GWI.

Much of the lagging therapeutic discovery in GI dysfunction related to GWI can be attributed to a limited understanding and lack of GI-specific model systems that provide deep cellular-level insights into multi-cellular physiology leading to GI motility or dysmotility. Bioengineering approaches aim to develop innovative models capable of conveying more accurate information about the cellular microenvironment. Bioengineering is a multidisciplinary field that combines principles and techniques from engineering, medicine, and biomedical sciences (such as cell and molecular biology, physiology, etc.). It focuses on solving intricate problems and developing innovative solutions to deepen our understanding of biological systems. Typical bioengineering strategies involve the combination of cells with biomaterials to create physiological functional tissue-engineered three-dimensional structures in vitro [19–26]. Nuances of GI bioengineering techniques including cell sources and scaffold compositions are reviewed comprehensively elsewhere [27]. Such in vitro bioengineering approaches enhance our understanding of cellular function in physiology and pathophysiology, complementing valuable insights gained from animal models and human clinical trials.

The goal of this review is to highlight in vivo models of GI dysfunction in GWI, as well as the emerging field of bioengineering within the GI domain. We conclude with thought-provoking future perspectives, challenges, and opportunities for bioengineered approaches specifically focused on studying GI dysfunction in GWI, with the hopes of moving therapeutics forward in GWI-related GI dysfunction.

## Toxic exposures and the origin of GWI

Gulf War personnel were exposed to a wide range of chemicals, including but not limited to 5 major classes of pesticides such as organophosphates, carbamates, N,N-Diethyl-3-methylbenzamide (DEET), and pyrethroids (permethrin). Other potential toxic exposures include sarin gas, depleted uranium, and emissions from oil well fires; however, their contribution to GWI is less known [4]. The US military provided personal use pesticides like DEET, commonly applied topically, and permethrin, sprayed onto uniforms, to combat arthropod-borne infectious diseases. However, documentation of individual exposure was relatively inconsistent [4, 9]. Organophosphates were another subset of pesticides that were used as sprays, powders, foggers, pest strips, and fly baits [28]. Another Gulf War-related exposure involved pyridostigmine bromide administered orally as a pre-treatment for nerve agents, particularly sarin gas. These exposures have been implicated as a major causation for GWI [29–31]. Studies have shown that combined exposure to DEET and/or permethrin with pyridostigmine bromide is known to exhibit synergistic effects even at low doses [32, 33]. Individually, these chemicals demonstrably infiltrate the CNS and peripheral nerves [17]. Organophosphates and pyridostigmine bromide also act as acetylcholinesterase (AChE) enzyme inhibitors, which terminates the action of the neurotransmitter, acetylcholine (ACh) [34]. It has been well reported that AChE enzyme inhibitors are associated with neuroinflammation of the CNS, consequently leading to classic signs such as diarrhea, constipation, muscle weakness, and abdominal pain classified under gut motility dysfunction and FGIDs [35–44]. Acute toxic exposure to AChE enzyme inhibitors has a predictable systemic effect that depends on the hyperactivity of cholinergic nerve receptors. While chronic effects of AChE enzyme inhibitors have also been linked to negatively impact memory, attention, and long-term brain function within rat models, indicating their potential to inflict neurobehavioral dysfunction [45, 46]. However, the absence of acute toxicity resulting from AChE enzyme inhibition has suggested that GWI is related to an underlying chronic neuroinflammatory disorder rather than an acute response to the direct inhibition of AChE enzyme-receptors [47]. Regardless, the main symptoms arising from impaired central and peripheral cholinergic function due to AChE enzyme inhibition are similar to those reported by afflicted Gulf War veterans, such as fatigue, musculoskeletal, cognitive, GI, and sleep problems [48, 49].

## GI dysfunction in the GWI clinic

Soon after the Gulf War, deployed veterans began experiencing chronic and unexplained conditions, as well as symptom clusters that defied conventional medical and psychiatric explanations, including standard laboratory tests. One of the challenges faced by clinicians during the post-Gulf War era was establishing criteria to diagnose Gulf War veterans due to these self-reported symptoms not representing any unique pattern of illness. Over time, there was an evolution in clinical definitions aimed at characterizing these predominantly unexplained symptoms, eventually referred to as GWI among Gulf War veterans. These clinical definitions include chronic multi-symptom illness (CMI) [8], the Kansas GWI definition [50], the Haley syndrome criteria [51, 52], and adaptations of these approaches. Most studies use the Kansas definition which encompasses symptoms falling into 3 out of 6 categories: fatigue and sleep problems, pain, neurologic and mood disorders, GI issues, respiratory problems, and skin symptoms categories [50].

GI complications are notably widespread and often co-occur with various other symptoms exhibited by veterans [53]. Intriguingly, while GI ailments are prevalent among veterans across different conflicts, they appear to be more frequent among Gulf War veterans and persist even after the cessation of war [54]. Findings from various cohort studies suggest that between 14 – 25% of Gulf War veterans report experiencing GI disturbances such as diarrhea, dyspepsia, abdominal pain and discomfort, constipation, nausea, IBS, and other functional GI disorders that originated during their deployment in the Gulf region and persisted upon return to the United States [8, 55–59].

The GI tract is a remarkable example of physiological complexity, encompassing many interactions and systems that are essential for proper functioning. Understanding the complexities of GI disorders has the potential to significantly advance the development of effective therapeutic approaches. In the subsequent sections, we provide an overview of the physiological mechanisms governing GI function in motility and their relevance to understanding GI disorders within GWI.

## GI neuroimmune crosstalk in motility in homeostasis and GWI

### Physiology at homeostasis

Normal intestinal function relies on the mechanics of GI motility to enable proper digestion, nutrient absorption or excretion, and water absorption [60]. Disruptions in intestinal motility can manifest as significant GI disorders including chronic constipation and diarrhea [61]. The intricacies of GI motility have been comprehensively reviewed by various experts in the field, and the interested reader is encouraged to refer to these reviews for a more profound understanding of GI physiology [62–66]. The gut-brain axis has also grown interested in its involvement in GWI reviewed by Slevin et al. [67], but in this review, we aim to introduce key cellular players and their roles in integrating neuro-immune communication that contributes to GI motility, highlighting how they are disconnected in GWI.

#### Smooth muscle

Motility in the GI tract is achieved through peristalsis, a mechanism characterized by waves of contractions that propel food or waste forward. This crucial function involves the coordinated shortening of the longitudinal smooth muscle cells and relaxation of the circular smooth muscle cells, resulting in intestinal expansion [68]. These intestinal smooth muscle cells respond to rhythmic impulses from motor neurons within the enteric nervous system (ENS), which is located within the GI tract itself. Ultimately, the smooth muscle layers in the gut play a pivotal role in facilitating motility, mixing, and propelling intraluminal contents, thereby enabling efficient digestion of food, progressive absorption of nutrients, and eventual evacuation of waste [69].

#### ENS

The ENS is an intricate network that regulates motor function, mucosal permeability, immune function, and endocrine secretion [70]. This interconnected web of sensory, motor, and interneurons heavily relies on cholinergic stimulus primarily utilizing the neurotransmitter ACh.

Motor nerves within the gut innervate both the circular and longitudinal smooth muscle layers (muscularis externa) and are responsible for stimulating smooth muscle force production through neurotransmitters. The main subtypes of motor nerves directly involved in gut motility are excitatory and inhibitory motor nerves, which depend on neurotransmitters such as ACh, vasoactive intestinal peptide, nitric oxide, and substance P. These motor nerves are predominantly located within the myenteric plexus, a network of nerves and glia situated between the smooth muscle layers in the muscularis externa.

Complex regulatory signal processing occurs both independently as well as in coordination with the CNS via the gut-brain axis to maintain homeostasis in the gut and other bodily systems. Disruptions to this neurological regulation have detrimental effects on gut health [71]. Abnormal functioning of excitatory and inhibitory motor nerves has been implicated in GI motility dysfunction observed in animal models [72, 73].

#### Muscularis macrophages within the gut

**Fig. 1:**
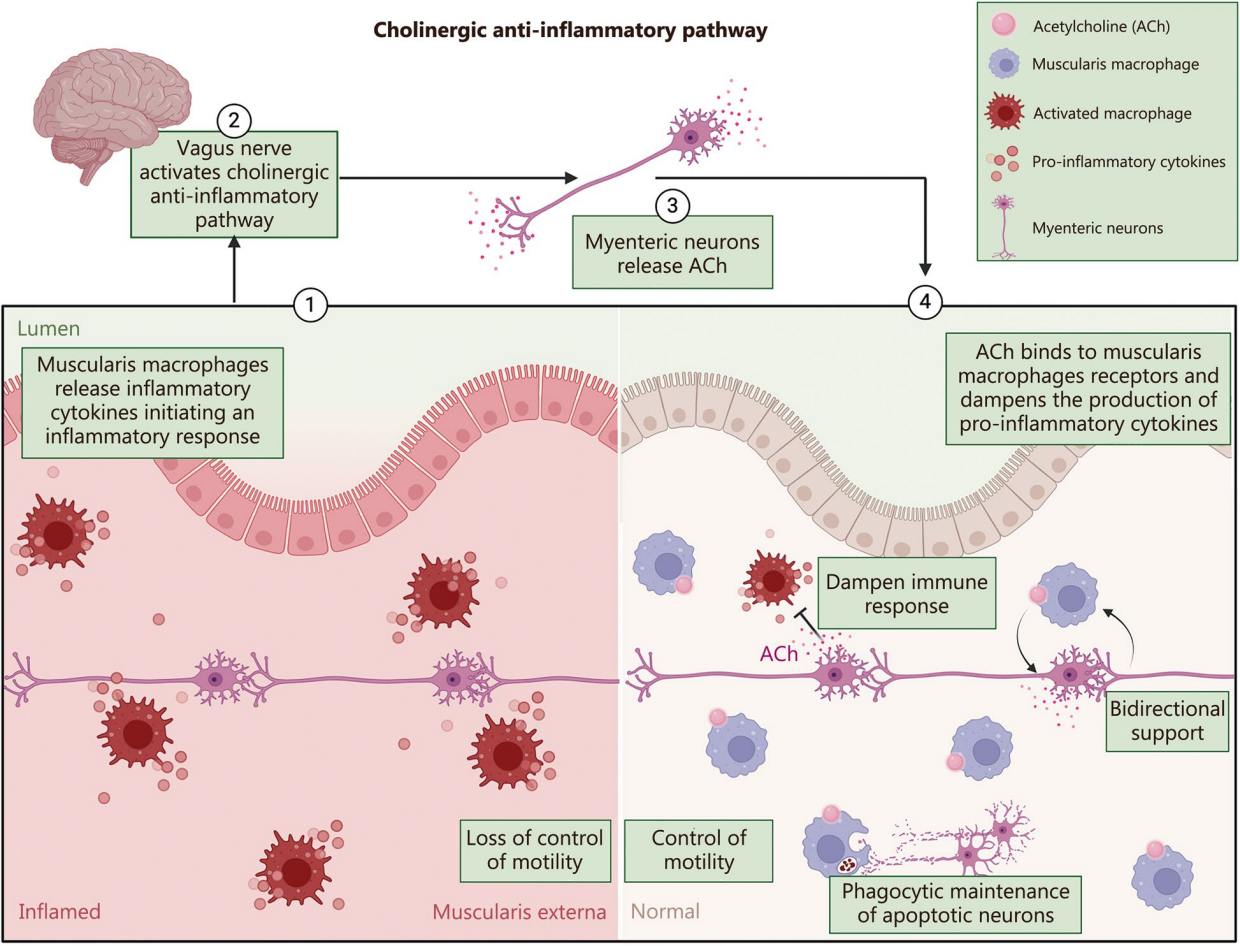
Muscularis macrophages interact with enteric neurons to maintain homeostasis within the gut. ① Muscularis macrophages become activated and release pro-inflammatory cytokines in response to inflammatory triggers ultimately leading to a loss of control of motility. ② The vagus nerve can stimulate enteric neurons and trigger an increased release of ACh. ③ This increase in ACh causes activated muscularis macrophages to return to a state of homeostasis where they offer bidirectional support and communicate directly with enteric neurons. ④ Muscularis macrophages even participate in the phagocytosis of surrounding apoptotic neurons. Ultimately with a balanced support of muscularis macrophages and enteric neurons, this improves the control of motility

Another aspect of the ENS is its ability to interact with the local immune system bidirectionally, maintaining homeostasis or contributing to pathological progression during infection and inflammation [74, 75]. From a perspective of gut motility, muscularis macrophages located within the smooth muscle layers and myenteric plexus of the gut, provide bidirectional support for the survival and differentiation of enteric motor nerves **(**Fig. 1**)**. Enteric neurons closely interact with muscularis macrophages and produce colony stimulating factor-1 (CSF-1), which in turn promotes their survival. Similarly, muscularis macrophages support neuronal function through the secretion of bone morphogenetic protein 2 (BMP2), thereby ensuring proper neuronal function and gut motility under normal conditions [76]. Muscularis macrophages exhibit a fascinating response to ACh released from stimulated enteric neurons via the vagus nerve, leading to a reduction in the production of pro-inflammatory cytokines [77]. Furthermore, these macrophages play a role in maintaining ENS balance by engulfing apoptotic neurons, and supporting the turnover of neurons [78].

### GI dysmotility and associated pathophysiology in GWI

During the onset of symptoms associated with GWI within the gut, particularly involving motility dysfunction through functional GI disorders, there can be a synergetic effect on the cellular mechanisms responsible for coordinating gut movement. Research has shown that exposure to agents related to Gulf War activities plays a role in activating macrophages and causing neurodegeneration of the ENS [15–17].

**Fig. 2:**
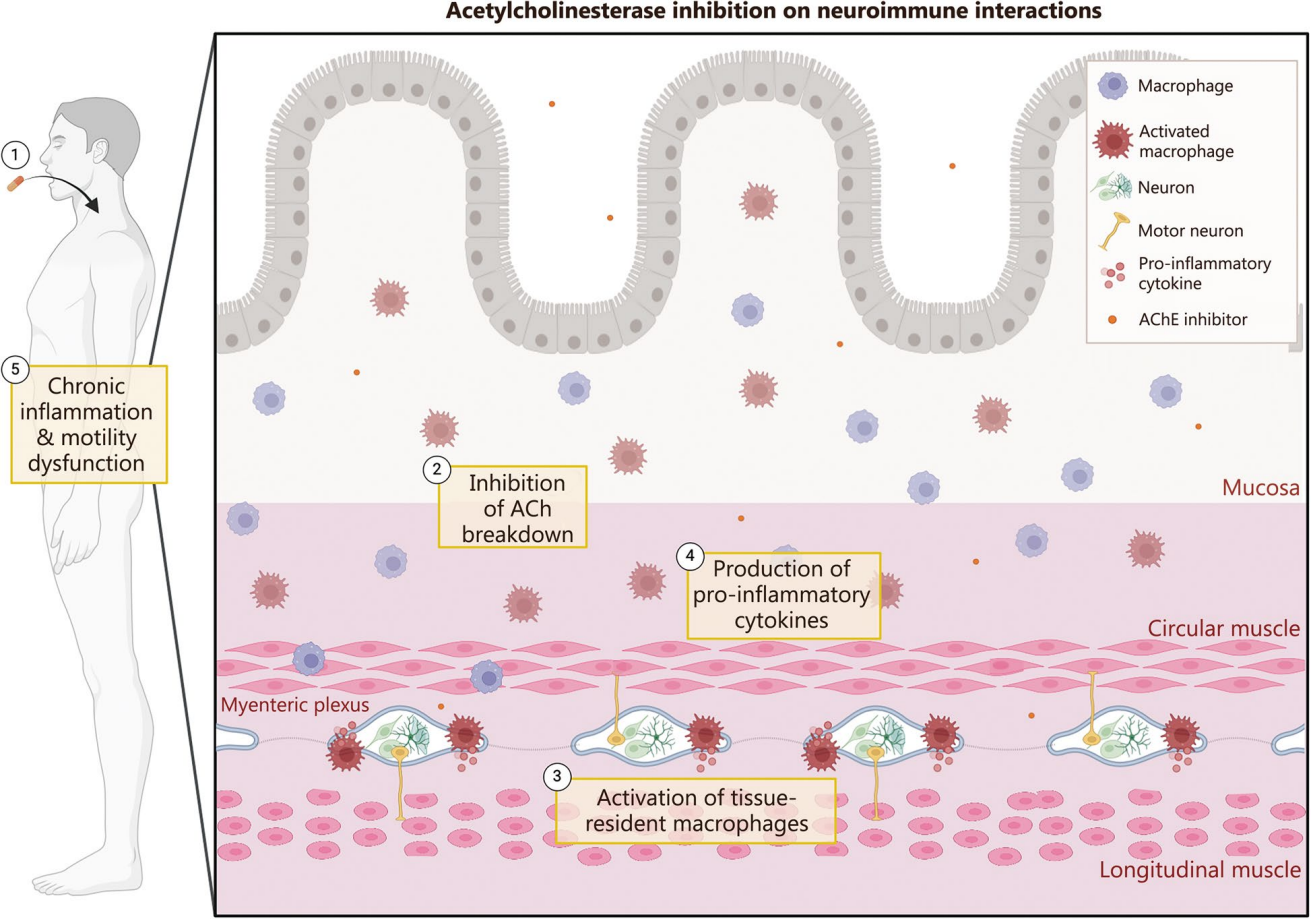
A summary of the effects of AChE inhibition on neuroimmune interactions. Once ingesting pyridostigmine bromide (PB), this incites an inhibition of ACh breakdown leading to a buildup of ACh. Tissue-resident muscularis macrophages become activated and trigger an inflammatory response leading to the release of pro-inflammatory cytokines. This inflammation present near enteric neurons can be classified as enteric neuroinflammation and leads to gut motility dysfunction, present in GWI. AChE acetylcholinesterase, ACh acetylcholine, GWI Gulf War Illness

The Gulf War agents primarily belong to a class of AChE enzyme inhibitors, which function by inhibiting the breakdown of the neurotransmitter–ACh. This inhibition leads to an accumulation of ACh near cholinergic receptors. Acute toxic exposures result in decreased responsiveness to ACh due to cholinergic receptor desensitization seen in both muscarinic ACh receptors [79–81] and nicotinic ACh receptors [82–84], a phenomenon widely observed in nerves within the CNS [85–89]. Systemically, ACh plays a vital role in dampening inflammation by suppressing pro-inflammatory markers and regulating immune cell activity [90, 91]. Conditions such as chronic inflammation or neurodegenerative diseases exhibit decreased ACh activity [92], altering immune modulation and disrupting the balance between pro-inflammatory and anti-inflammatory processes in the gut [93, 94]. Similar neuroinflammatory phenomena are noted secondary to exposure to Gulf War agents within the ENS [12, 16, 17]. When neuroinflammation extends to both nervous and immune components of the enteric nervous system, it results in enteric neuroinflammation that affects the coordination of gut motility at all levels discussed in Sect. "Physiology at homeostasis" involving various cellular players [15–17]. Figure 2 illustrates the mechanism through which inflammation is caused by the inhibition of AChE. While this review primarily focuses on the pathophysiology of the cellular components responsible for GI motility, namely smooth muscle cells, enteric motor nerves, and muscularis macrophages, it is crucial to acknowledge the significant implications of the gut microbiome on overall GI tract health and function through its interaction with the gut-immune axis. The gut microbiota plays a pivotal role in regulating the neuro-immune axis by influencing GI barrier function, immunity, and host protection [95], which ultimately impacts gut motility. In inflammatory bowel diseases and other inflammatory conditions affecting the gut, dysbiosis in the gut microbiome impairs colonic motility and transit [96, 97], resulting in a reduction of microbial genes by 25% compared to healthy individuals [98]. Even in the context of GWI, veterans experiencing GI symptoms had an altered microbiome compared to those without GI manifestations [99]. Therefore, considering disruptions in gut motility necessitates careful consideration of alterations in the gut microbiome.

A comprehensive investigation into the physiology/pathology of GI disorders in GWI lays the foundation for targeted therapeutic interventions. Understanding the intricacies of GI manifestations in GWI, including alterations in gut microbiota, inflammatory responses, gut motility dysfunction, and dysregulated neural signaling, is crucial for developing effective treatment strategies. Defined comprehensions of these manifestations are necessary to facilitate targeted and efficacious therapeutic interventions. Therefore, bridging the gap between the understanding of GWI-GI pathophysiology and conducting therapeutic intervention studies can improve the management of GI-related symptoms and enhance the overall quality of life for Gulf War veterans. This next section explores current therapeutic interventions and their challenges in treating GI-related symptoms among Gulf War veterans.

## GI-related therapeutic intervention studies in GWI

GWI-related clinical trials have aimed to investigate the effectiveness of existing interventions in treating similar symptomatic manifestations in other conditions. A wide array of interventions including probiotics, supplements, cognitive behavioral therapy, exercise, medications, and anti-inflammatory drugs have been utilized to improve the overall quality of life for Gulf War veterans. Psychological and exercise interventions, including cognitive behavioral therapy and mindfulness techniques, focus on alleviating GWI symptoms such as pain, fatigue, and cognitive deficits without addressing underlying causes [100–104]. These methods provide moderate relief across various symptom domains. In contrast, medications like doxycycline [105], mifepristone [106, 107], and naltrexone [108] showed limited effectiveness while potentially causing adverse effects or showing no significant improvement. Nutritional interventions like Coenzyme Q10 [109, 110] yield mixed results, whereas ongoing studies explore brain stimulation [111–115], and botanical compounds [116–119] for managing GWI symptoms, encompassing cognitive deficits, chronic fatigue, and GI issues.

Although these interventions strive to improve the overall quality of life for Gulf War veterans, they fall short in addressing the specific targeting of GI symptoms associated with GWI. This presents a significant challenge in managing this aspect of the condition. The complex nature of these symptoms, including diarrhea, constipation, abdominal pain, and IBS, necessitates targeted interventions tailored to meet the unique needs of Gulf War veterans. Developing effective interventions for these symptoms remains a crucial yet unmet requirement in the comprehensive management of GWI.

Currently, there is a limited number of clinical interventions available for alleviating GI symptoms in Gulf War veterans. One study assessed the effects of Rifaximin, an antibiotic, on a subpopulation of Gulf War veterans diagnosed with IBS, aiming to restore gut microflora. However, this intervention did not demonstrate any in relieving symptoms such as abdominal pain, bloating, stool urgency/frequency/consistency [120]. Another study investigated carnosine, an endogenous antioxidant believed to aid in the elimination of prolonged reactive oxygen species (ROS). Baraniuk et al. [121] found some improvements in GI symptoms including diarrhea. Probiotics have also been examined as a treatment option for GI symptoms. Studies utilized Visbiome, which contains 8 strains of bacteria and has shown efficacy in improving ulcerative colitis [122, 123], however significant results were not observed when it came to alleviating IBS symptoms in comparison to control groups [124]. Dietary interventions involving low-FODMAP (Fermentable Oligo-, Di-, Mono-saccharides, and Polyols) diets for IBS management among Gulf War veterans have been explored; however, no published results are currently available [125]. Nevertheless, other studies using a low-FODMAP diet have demonstrated improvements in alleviating abdominal pain, bloating, and diarrhea [126]. While there are positive outcomes associated with low-FODMAP diets, there are concerns regarding the long-term impact on intestinal microbiome composition, metabolism, and nutritional status. A recent study has commenced investigating the effect of butyrate, a short-chain fatty acid, on alleviating GI symptoms (such as diarrhea, pain, and constipation) through modulation of the gut microbiome and anti-inflammatory effects. Although still in its early stages, this research is anticipated to yield similar benefits in managing GI symptoms to those seen in previous studies on IBS [127].

These clinical interventions offer a significant advantage by directly engaging with the study population to improve patient care. However, they pose unique challenges due to the intricate nature of gut physiology and the multifactorial etiology of GI disorders, particularly in GWI. There is an evident disparity between GWI research and clinical trials as most studies focus on overall patient health, while only a fraction specifically addresses issues related to the GI tract. Emphasizing the importance of the GI tract in GWI is crucial for several reasons. Firstly, the GI tract serves as an important link between the external and internal environments. It plays a significant part in nutrient absorption, waste secretion, immune responses, and microbial balance, making it a key determinant of overall patient health and well-being. Furthermore, neglecting the GI tract may result in incomplete assessments of the illness, delaying the development of effective treatment approaches.

To address these challenges, researchers are increasingly turning to complementary approaches, including animal models and bioengineering techniques for both the discovery of therapeutic avenues and gaining better insights into pathophysiology. Animal models serve as invaluable tools for investigating disease pathogenesis, testing hypotheses, and evaluating therapeutic interventions within in vivo systems. Concurrently, bioengineering models provide controlled microenvironments to simulate complex physiological conditions ex vivo, facilitating targeted studies on specific aspects of GI function and therapeutic responses. Combining bioengineering models and animal studies with clinical trials can address the limitations intrinsic to human-based investigations, offering comprehensive and multi-faceted approaches to enhance our understanding of GWI-GI disorders and improve clinical outcomes. The following sections explore advancements in animal models and bioengineering in elucidating the complexities of GI physiology and pathology of GWI.

## In vivo models of GI dysfunction in GWI

The assessment of the role of exposure factors in the development of GWI is challenging due to the limited availability of robust data regarding the precise dosages, frequencies, and durations of these exposures during combat. Furthermore, the complexity arises from the presence of multiple toxicants and unknown interactions between them in the war theater. Additionally, uncertainties remain regarding the effects of repeated exposures to the same toxicant under stressors associated with war and deployment.

The early comprehension of the multifactorial origins of GWI has been largely shaped by preclinical research, with rodent models playing a pivotal role. These models have been used to investigate mechanisms underlying GWI symptoms afflicting various bodily systems such as the brain, gut, muscles, and blood. Initial studies on toxic exposures leading to neuroinflammation in the CNS were instrumental in establishing routes and modes for studying GI dysfunction.

Recently, there has been an increased focus on the GI symptoms related to GWI (Table 1) [15–17, 128]. Although relatively new, a modified animal model has emerged that specifically targets the CNS and exposes 5-week-old male and female mice to oral pyridostigmine bromide (PB) for 7 d. This 7-day exposure led to acute alterations in colonic motility and structure in both male and female mice. These were followed by increases in enteric neuroinflammation, glial reactivity, and dysfunctional anatomy of the colon in a sex-dependent manner [16, 17]. Additionally, this study successfully identified key differences between male and female mice upon PB exposure. For example, while both sexes experienced alterations in colonic motility, males exhibited a greater increase whereas females initially displayed lower levels which then progressed into higher levels of colonic motility at day 7. Identifying sex-dependent differences related to GI symptoms can significantly contribute to the development of more effective and personalized treatments. These variations in GI symptoms may potentially arise from hormonal influences, genetic predispositions, or underlying physiological conditions [129]. Age also plays a significant role in gut health due to its substantial effects on immunity, gut motility, and microbial composition [130].

**Table 1:** Summary table of gastrointestinal (GI)-related experimental designs of Gulf War-related rodent models

**Application**	**Species**	**Experimental design**	**Proposed mechanism/results**	**Limitation**s	**Reference**
Pre-clinical murine model	5-week-old C57BL/6 male and female mice	Mice received either 9 μg/ml or 90 μg/ml of PB (oral) for 7 d	This study found that PB altered glial activity and neural survival within the colon. There were also differences in motor functions between male and female mice	While this study looks at both the effects of PB on the brain and the gut in male and female mice, research in older mice would better replicate the aging population of Gulf War veterans	[16, 17]
Pre-clinical murine model	34-week-old C57BL/6 male mice	Mice received 2 mg/kg of PB (oral) for 7 d	This study found that PB altered contractile patterns with increased signs of neuroinflammation. Cholinergic neurons were seen to be decreased with a synonymous increase of CD40^+^ pro-inflammatory macrophages	The study only includes male mice and does not include chronic effects of PB exposure	[15]
Pre-clinical murine model	10-week-old C57BL/6J male mice	Mice received 2 mg/kg of PB (oral) + 200 mg/kg of PM for 2 weeks, 3 weekly	An obesity phenotype in GWI-induced mice can worsen GI symptoms and neuroinflammation associated with glial activity	The study used a moderate sample size (5 – 6) per treatment group, although the effect sizes of the different treatments were considerably large. The study also lacks mechanistic studies of GI motility in response to chemical exposures	[128]

*GWI* Gulf War Illness, *PB* pyridostigmine bromide, *GI* gastrointestinal

A study done by Gwini et al. [131], suggested that there is a need to consider the relationship between persistently increasing symptoms and long-term morbidity, particularly in aging veterans. Our research group has addressed this concern by subjecting 32- to 34-week-old male mice to PB for a duration of 7 d, evaluating the effect of PB exposure on the repair aspect of the colon of older mice [15]. The results revealed an increase in enteric neuroinflammation, reduced colonic motility, decreased population of enteric neuronal stem cells (ENSCs), and smooth muscle hypertrophy. ENSCs play a crucial role within the GI in maintaining and repairing normal functionality of mature neurons [132]. These findings highlight how PB exposure can exacerbate symptoms as patients get older and emphasize the diminished repair function of ENSCs as a contributing factor to the enduring effects of GWI after initial exposure. Another study has examined obesity as another contributing factor to the exacerbation of GI-related symptoms in GWI. Furthermore, this study examined how a Western-style diet can potentially aggravate GI-related GWI symptoms, considering the potential changes in patients’ diet and lifestyle as they age. Results indicated an elevated presence of persistent GI and neuronal inflammation within obese phenotype in individuals with GWI [128].

The complexity of the human GI system cannot fully be replicated by mouse models. Rudimentary differences between the human and mouse gut anatomy, microbial composition, and physiological responses lead to variations in the development of GWI symptoms [129]. Therefore, while rodent models have been crucial in understanding the complex mechanisms associated with the pathophysiology of diseases, caution must be practiced when applying these results to the etiology of human GWI. An emerging trend in mouse models involves humanizing mice with functional human immune systems through engraftment of human cells. Studies have successfully transplanted human gut microbiota and epithelial cells into mice to recreate aspects of human GI physiology and pathology within an animal model [133]. For instance, humanized mice have been instrumental in studying the role of gut microbiota in the development of diarrhea and constipation, allowing researchers to investigate the underlying mechanisms and identify potential therapeutic targets [134, 135]. Moreover, humanized mouse models can be used to test the efficacy of novel interventions, including probiotics, prebiotics, and dietary supplements, in alleviating GI symptoms [136–138]. In the context of GWI-GI, future studies could include incorporating humanized mice to assess immune alterations within the GI tract. Furthermore, elevated cytokines found in Gulf War clinical studies like tumor necrosis factor (TNF) [99] could be added to monitor symptom progression, evaluate treatment efficacy, and elucidate underlying mechanisms, ultimately enhancing relevance and translational potential in GWI-GI disorder research.

Ultimately, GWI has been shown to impact the normal functionality of the gut in both young and older animal models; however, there remains a dearth of research on numerous contributing factors that can exacerbate symptoms, thereby leading to significant concern regarding the treatment of aging patients and identification of specific biomarkers for GI symptoms to facilitate more clinical trials/interventions. In addition to age increase, animal models have reached limits of how in-depth researchers can understand sub-cellular mechanics. In the next section, we will explore how in vitro research can be used to dive deeper into the cellular interactions within the pathophysiology of GWI.

## The potential of bioengineered models with GI focus for GWI

The field of in vitro models has undergone significant advancements over the years, enabling the study of a diverse range of diseases, including GI pathology. However, when it comes to GWI, there is a scarcity of in vitro studies specifically focused on GI symptoms. In this section, we will examine the existing in vitro models that concentrate on GWI and provide perspective on future ideas for in vitro models in the GWI-GI context.

Several in vitro models have focused on identifying neurotoxicity caused by Gulf War agents within CNS neurons of humans and rats [139–143]. While these studies have revealed associations between inflammation, increased apoptosis, and persistent neurodegeneration, shedding light on direct and indirect priming effects resulting from exposure to GW agents on specific cell types; they do not directly explore enteric nerves and their effect on GI motility and physiology.

Our research group has successfully isolated various cell types including colonic smooth muscle cells (cSMCs), ENSCs, and bone marrow-derived macrophages from mouse models with GWI. We conducted a study to investigate the effects of acute exposure to pyridostigmine bromide on these cell types after 7 d. Specifically, we explored how pyridostigmine bromide exposure affects the proliferation and characteristics of ENSCs. Our findings revealed that the ENSCs exhibited reduced proliferation rates and lower levels of stem cell markers (*Sox2*, *Sox9*, *Ngfr*) [15]. Additionally, hypertrophy was observed in the musculature of the colon. Notably, our group was the first to demonstrate that exposure to pyridostigmine bromide alters enteric neural stem cell proliferation and induces phenotypic changes in colonic smooth muscle cells [15].

The complexity of GWI, which involves a wide range of GI symptoms and cellular interactions, necessitates the use of more sophisticated in vitro models to evaluate functional outcomes resulting from cell–cell interactions. In related chronic inflammatory diseases with GI symptoms [144–146], various in vitro models such as co-culture organoids systems, gut-on-a-chips, and bioengineered tissues have been employed to investigate cellular interactions.

### Organoid models

Organoids, also known as 3D cell clusters, have gained popularity due to their ability to mimic physiological properties and facilitate disease modeling [147]. Specifically, GI organoids have been successfully generated from adult human intestinal stem cells and pluripotent stem cells, forming tissue-like structures with multiple layers including the intestinal epithelium, mesenchymal and intestinal stem cells, enterocytes, and secretory cells [148]. With this approach, stem cells are embedded in extracellular matrix (ECM)-rich hydrogels such as Matrigel [149, 150]. The culture medium containing growth factors is critical for GI organoid expansion and may vary slightly between murine and human organoids, but generally include molecules like Wnt/R-spondin, Bone morphogenetic protein (BMP)/Noggin, and Epidermal growth factor (EGF). Moreover, GI organoids can be tailored towards distinct sections of the gut such as the small intestine [151, 152] or colon [153–155]. Additionally, these organoids have evolved to incorporate co-cultures with immune cells. For example, Kakni et al. [156] developed a microwell-based coculture system of intestinal organoids and RAW264.7 macrophages supplemented with 2% Matrigel. Upon treatment with TNF-α in these co-cultures of organoids and immune cells, cytokine secretion profiles associated with inflammatory responses [including interleukins, Regulated on Activation, Normal T Expressed and Secreted (RANTES), and interferon- γ (IFN-γ)] were observed resembling aspects of acute and chronic inflammation. Biton et al. [157], on the other hand, found that co-culturing intestinal organoids with polarized T helper cells or IL-17 increased differentiation of Lgr5^+^ ISCs into goblet cells, Paneth cells, and enteroendocrine cells. Other co-culture systems have investigated the impact of IL-22, IL-2, and IL-13 on the proliferation and differentiation of intestinal organoids, demonstrating that immune cell-derived cytokines and the microbiome can influence the differentiation of intestinal epithelial cells [157–161]. Additionally, these co-culture studies have been utilized to model host–pathogen interactions in response to bacteria [162, 163], viruses [164], and parasites [165]. Although modeling epithelial layers of the gut has been relatively straightforward using the organoid approach, comprehensive investigations often neglect to evaluate GI motility with these approaches. Nevertheless, patient-derived co-culture organoid models remain valuable for studying inflammatory responses in pathogenic conditions by incorporating GW toxic exposures, as well as relevant biomarkers associated with GWI. Remarkably, functional ENS components have been incorporated into organoids featuring a diverse population of neurons capable of inducing smooth muscle contractions through low voltage stimulation [166]. Additionally, Park et al. [167] successfully generated colonic organoids from human embryonic stem cells that contained enteric neural crest stem cells along with mature nerves and blood vessels. Functional enteric nerves and smooth muscle can also be incorporated into GI organoids using cells derived from the three primary germ layers (enteric neuroglial, mesenchymal, and epithelial precursors) stemmed from human pluripotent stem cells [168]. Eicher et al. [168] developed a 3-germ-layer human gastric organoid consisting of glandular epithelium surrounded by functional smooth muscle innervated by enteric neurons capable of regulating contractions. These advancements in intestinal organoids, which possess an intact enteric neuroimmune function, can be tailored to specific GI organs and a variety of diseases, including in the pathogenesis of GI-related GWI. They provide researchers with the opportunity to investigate functional and morphological changes occurring during human ENS-GWI development and progression, as well as explore neuroimmune interactions.

### Gut-on-a-chip

Another emerging bioengineering model for studying complex GI diseases is the gut-on-a-chip system. These models can integrate living human cells with mechanical and biochemical cues to mimic the physiological conditions of the gut [169]. The gut-on-a-chip enables real-time monitoring of cellular responses, including barrier function, immune interactions, and peristalsis-like movements, offering a dynamic and physiologically relevant platform for toxicology, pharmacology, and drug studies [170, 171]. A majority of these microfluidic devices are able to replicate the epithelial layer with mucus production and microbiota, including a vascular component to mimic the absorption and distribution of nutrients [169, 172, 173]. Additionally, these systems have expanded beyond just the epithelial layer; they now encompass ‘muscle-on-a-chip’ models that incorporate skeletal and smooth muscle engineering to achieve functional muscle contraction [174–177]. By incorporating engineered intestinal smooth muscles on-a-chip, these models can be tailored to study GWI-GI dysfunction and screen for GW toxicants. Furthermore, co-culturing various cell types including immune cells and neurons, could enhance the predictive power of preclinical studies by more accurately mimicking human physiology.

### Bioengineered intestines

Altered neural networks have been observed in pre-clinical models of GWI [178], as well as in aging individuals, which is also relevant for Gulf War veterans [179, 180]. Bioengineered intestines have been developed within a collagen gel that exhibits physiologically relevance and functional capability to contractile responses. These bioengineered intestines incorporate smooth muscle, neural progenitor cells, glial cells, and mature differentiated nerves [19, 20, 24–26]. Therefore, bioengineered muscle could be utilized as a therapeutic approach specifically targeting GI symptoms related to motility dysfunction. On a more fundamental level, these bioengineered intestine models can be employed to study pathological and physiological cellular interactions predominantly present in the outer layer of the colon (as opposed to organoids). Moreover, in the context of GWI, these models can be exposed to Gulf War toxicants; alternatively, tissues donated from Gulf War veterans can be incorporated into these models for drug screening and disease modeling.

## Future directions and challenges for bioengineering within GWI

Bioengineering has the potential to revolutionize crucial aspects of medical research, disease pathophysiology, and drug screening development, thereby advancing clinical trials. The precision afforded by bioengineering in genetic manipulation, high-throughput screening, and personalized medicine enhances the efficiency of drug discovery and development processes. Furthermore, bioengineered platforms can significantly reduce reliance on animal testing, while providing more accurate representations of human biology. These innovations can accelerate the understanding of disease pathology and mechanisms, facilitate the identification of potential drug candidates, and enable the development of tailored treatments. Ultimately, this will enhance the effectiveness and safety of interventions tested in clinical trials.

Human patients exhibit significant variations in diet, lifestyle, and genetic makeup, among other criteria, all of which can be implicated in neurological or GI dysfunction. Animal models are important contributors to understanding human physiology by offering similar biological processes, disease modeling, genetic contributions and manipulations, immunological responses, as well as translational research opportunities. However, animal physiology does not fully replicate human physiology, which limits the capacity to effectively test current experimental treatments such as CRISPR-based editing that are specifically tailored to the human genome, or even to accurately predict human toxicity [181]. Controlling this intrinsic variability could provide more meaningful and significant results in the study of human diseases and evaluation of experimental, genetically derived treatment while also facilitating the investigation of novel solutions posited by ongoing research.

**Fig. 3:**
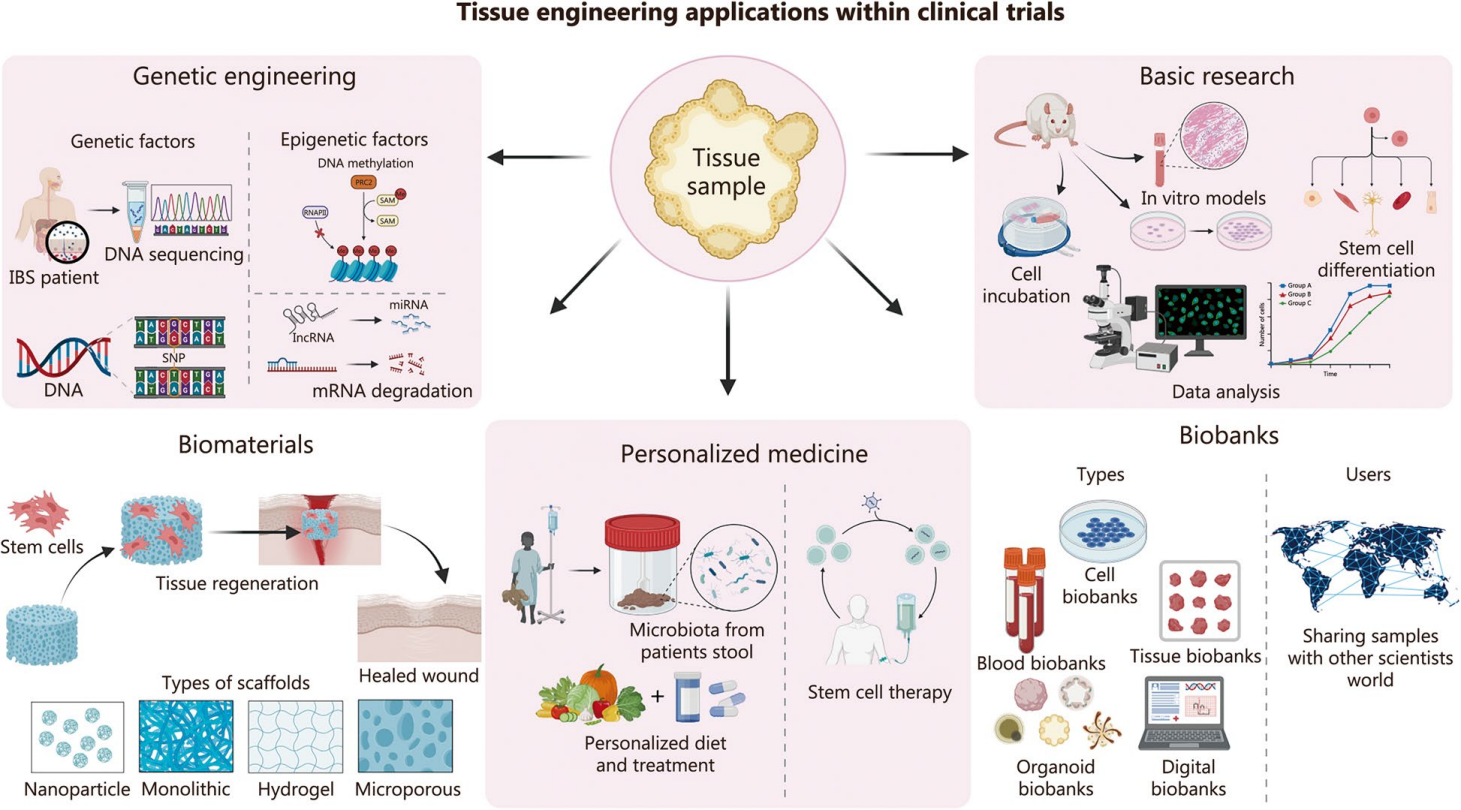
Various applications of tissue engineering that can be employed through the use of genetic engineering, basic research, implementation of biomaterials, stem cell therapy for personalized medicine, and biobanks which serve to store clinical, demographic, and genetic data. Ultimately tissue engineering can be tailored to each patient and can offer multiple avenues of personalized therapy

Moreover, the progression of clinical trials often entails extensive timelines, with only a fraction successfully overcoming initial checkpoints. This is where the implementation of bioengineering can enhance early-stage clinical trial advancement through genetic engineering, integration of biomaterials, utilization of biobanks, and a focus on personalized medicine (Fig. 3). For instance, within IBS models, bioengineering offers the exploration of genetic and epigenetic influences through whole genomic sequencing [182, 183], as well as transcriptomics for identifying biomarkers [184]. Epigenomic mechanisms influenced by intricate environmental factors may affect the genome, leading to potential implications for IBS [183]. Understanding the interactions between genetic, epigenetic, environmental, and peripheral factors in IBS provides valuable insights into potential treatment approaches. Such insights could be utilized for the parallel and multifaceted nature of GWI, offering promising avenues for clinical intervention.

Additionally, biobanks are being utilized to screen for biomarkers and omics but can also use comprehensive lifestyle and health-related information, biological measurements, and even brain imaging data to overcome extreme heterogeneity of phenotypes and allow for extensive data analysis on subpopulations [185]. Noteworthy examples of GWI biobanks include the Boston Biorepository, Recruitment, and Integrative Network (BBRAIN), Gulf War Era Cohort and Biorepository (GWECB), and Gulf War Veterans’ Illnesses Biorepository (GWVIB) [186–190]. Yates et al. [191] established a bank of human induced pluripotent stem cells (hiPSCs) from GWI veterans and unaffected individuals stored in BBRAIN. They further developed cerebral organoid cultures capable of differentiating into various cell types including neurons and glia. Another similar study found altered mitochondrial dynamics, decreased neuronal activity, and possible links between predispositions to GWI in some veterans [192]. The GWECB was specifically designed to investigate genetic and environmental associations with GWI by collecting blood samples from over 1000 Gulf War veterans for its repository [190]. Vahey et al. [193] analyzed single nucleotide polymorphisms (SNPs) near *ACHE*, *BCHE*, and *PON1* genes using samples from GWECB, revealing associations between *PON1* gene variants with pyridostigmine bromide exposure in severe cases of GWI and respiratory symptoms related to GWI. Ultimately, these biorepositories can help to advance research by providing biological samples from Gulf War veterans and future studies can be focused on exploring associations between GI symptoms related to GWI exposure variants.

Bioengineering models could also provide the capacity to study individual systems to assess their response to specific drugs or treatments in isolation, a feat that is currently unachievable in vivo. GWI contains a multitude of variables that simultaneously impact multiple organ systems, rendering it increasingly complex to identify precise interactions. Meanwhile, through bioengineering, it’s possible to control these variables, facilitating the sequential testing of individual factors (Fig. 3).

In this regard, the versatility of bioengineering is highly desirable. It enables the deliberate exclusion of confounding interactions commonly observed in biological subjects, such as the neuroimmune crosstalk in GWI. This facilitates more precise identification of vital receptor stimuli and thus leads to a more effective drug design. Also, the ability to use human-relevant tissues with reduced ethical concerns provides an opportunity to test a wider variety of potential treatments in a more relevant context than murine models can offer. In summary, bioengineering holds immense potential and promises extensive applicability in clinical and academic research, enhancing the utilization of human cells as experimental samples for obtaining more relevant, robust, and time-efficient results.

Bioengineering models, while essential for studying pathogenesis, exhibit limitations that warrant consideration in scientific endeavors. Notably, these models serve as a valuable tool for improving early-stage clinical studies, to evaluate treatment efficiency and possible adverse effects. However, these models cannot substitute phase 3 trials required for final drug approval. Phase 3 trials determine the effectiveness and safety of treatments in large patient populations with diverse genetic and biological factors. Additionally, bioengineering models are unable to fully mimic the intricate interactions and outcomes observed in the real-world environment on a larger scale, leading to disparities between observed behaviors and actual disease progression. The absence of systemic and genetic factors such as immune responses and hormonal regulation further hinders their ability to capture the complete spectrum of pathogenic mechanisms that are seen in sex-dependent differences. While bioengineering models can examine specific components like the immune system or enteric nervous stem within the GI tract, replicating the entire colon incorporating vast cellular interaction options becomes increasingly complex. Additionally, the translation of bioengineering techniques into human patients presents emerging challenges in ethics and policy. Central to these concerns is the notion of informed consent, whereby patients must fully comprehend the experimental nature of the treatment along with its potential risks, benefits, and long-term implications. A comprehensive regulatory plan is also necessary to evaluate any translational and transplantation potential of bioengineered human tissue equivalents, with an emphasis on safeguarding patient safety.

Despite its limitations, the future of bioengineering models in the GI tract holds promise for transformative advancements that can be applied to GWI-GI-related symptoms. Innovations in organoid models, organ-on-a-chip, and bioengineered intestine technologies will enable more physiologically relevant GI models by incorporating features like peristalsis and microbial interactions. The integration of microbiome and neuroimmune dynamics, personalized medicine approaches, and advanced biomaterials could enhance the anatomical accuracy and functionality of GI models. Additionally, the prospect of functional GI tissue engineering and multi-organ models signifies potential breakthroughs in therapeutic interventions and systemic understanding. Collectively, these bioengineering approaches aim to play a pivotal role in advancing research on drug development and assessment, disease modeling, as well as personalized treatments for GI disorders reported in GWI.

Through the application of techniques such as genetic engineering, biomaterials, biobanks, and personalized medicine, the integration of tissue engineering in GWI clinical trials could be a critical step forward in addressing this complex condition with multiple symptoms, offering valuable insights that may extend to similar conditions as well. Furthermore, bioengineering can be employed alongside animal models, which provide a controlled environment for studying disease pathways and evaluating potential therapies before progressing to clinical trials. On the other hand, clinical trials provide invaluable data about the safety and efficiency of the treatment in human populations. However, traditional models have limitations, including time expenditure, ethical and regulatory considerations, and patient availability. Tissue engineering techniques possess unique capabilities to bridge these gaps by creating in vitro models that mimic human or human-equivalent tissue structures and functions. This enables researchers to tailor treatments according to individual needs and predict patient responses more accurately while informing future study designs involving animals and humans.

## Data Availability

Data sharing is not applicable to this article as no datasets were generated or analyzed during the current study.

## Abbreviations

ACh: Acetylcholine
AChE: Acetylcholinesterase
BBRAIN: Boston Biorepository, Recruitment and Integrative Network
BCHE: Butyrylcholinesterase
BMP2: Bone morphogenetic protein 2
CMI: Chronic multisymptom illness
CNS: Central nervous system
CRISPR: Clustered Regularly Interspaced Short Palindromic Repeats
CSF-1: Colony stimulating factor-1
cSMCs: Colonic smooth muscle cells
DEET: N,N-Diethyl-3-methylbenzamide
ECM: Extracellular matrix
ENS: Enteric nervous system
ENSCs: Enteric neural stem cells
FGIDs: Functional gastrointestinal disorders
FODMAP: Fermentable Oligo-, Di-, Mono-saccharides and Polyols
GI: Gastrointestinal
GW: Gulf War
GWECB: Gulf War Era Cohort and Biorepository
GWI: Gulf War Illness
hiPSCs: Human induced pluripotent stem cells
IBS: Irritable bowel syndrome
IL: Interleukin
ISCs: Intestinal stem cells
MMs: Muscularis macrophages
Ngfr: Nerve growth factor receptor
PB: Pyridostigmine bromide
PON1: Paraoxonase 1
ROS: Reactive oxygen species
Sox2: Sex determining region Y-box 2
Sox9: SRY-related HMG box gene 9
SNPs: Single nucleotide polymorphisms
TNF: Tumor necrosis factor
